# Optimal classifier for imbalanced data using Matthews Correlation Coefficient metric

**DOI:** 10.1371/journal.pone.0177678

**Published:** 2017-06-02

**Authors:** Sabri Boughorbel, Fethi Jarray, Mohammed El-Anbari

**Affiliations:** 1 Systems Biology Department, Sidra Medical and Research Centre, Doha, Qatar; 2 Laboratoire Cedric, CNAM, Paris, France; 3 Clinical Research Center, Sidra Medical and Research Center, Doha, Qatar; Tianjin University, CHINA

## Abstract

Data imbalance is frequently encountered in biomedical applications. Resampling techniques can be used in binary classification to tackle this issue. However such solutions are not desired when the number of samples in the small class is limited. Moreover the use of inadequate performance metrics, such as accuracy, lead to poor generalization results because the classifiers tend to predict the largest size class. One of the good approaches to deal with this issue is to optimize performance metrics that are designed to handle data imbalance. Matthews Correlation Coefficient (MCC) is widely used in Bioinformatics as a performance metric. We are interested in developing a new classifier based on the MCC metric to handle imbalanced data. We derive an optimal Bayes classifier for the MCC metric using an approach based on Frechet derivative. We show that the proposed algorithm has the nice theoretical property of consistency. Using simulated data, we verify the correctness of our optimality result by searching in the space of all possible binary classifiers. The proposed classifier is evaluated on 64 datasets from a wide range data imbalance. We compare both classification performance and CPU efficiency for three classifiers: 1) the proposed algorithm (MCC-classifier), the Bayes classifier with a default threshold (MCC-base) and imbalanced SVM (SVM-imba). The experimental evaluation shows that MCC-classifier has a close performance to SVM-imba while being simpler and more efficient.

## 1 Background

Data imbalance occurs when the sample size in the data classes are unevenly distributed [1]. Such situation is encountered in many applications of bioinformatics [2, 3] such as pre-clinical drug adverse event, diagnosis of rare diseases, classification of primary form of rare metastatic tumors, early prediction of medical events from time-series data, etc. Most standard machine learning algorithms work well with balanced training data but they face challenges when the dataset classes are imbalanced. In such situation, classification methods tend to be biased towards the majority class. These algorithms are inefficient in this case mainly because they seek to maximize a measure of performance such as accuracy which is no longer a proper measure for imbalanced data. Accuracy treats equally the correctly and incorrectly classified examples of different data classes. For example, consider a data set that has 10% positive class and 90% negative class. A naif classifier that always outputs the majority class label will have a high accuracy of 0.90. As the data imbalance is more pronounced, the evaluation of the classifier performance must be carried out using adequate metrics in order to take into account the class distribution and to pay more attention to the minority class. According to Haibo, learning from class imbalance can be divided into two approaches: 1) data-level strategies such as re-sampling or combinations and 2) algorithmic strategies such as cost-sensitive and boosting [4]. The former approach re-balances the class distribution by either oversampling the minority class or undersampling the majority class or by combing the two. The later approach seeks to learn more from the minority class by setting a high cost to the misclassification of this class. A number of metrics have been studied for the purpose of classifying imbalanced data [5–10]. Tables 1 and 2 describe some known metrics that have been studied in this context. In this paper we address the optimality and the consistency of binary classification based on MCC metric. Similar to the approach presented by Oluwasanmi et al., we derive the optimal form of the Bayes classifier using the Frechet derivative of the MCC metric [6]. We prove the consistency of the proposed algorithm following the theoretical framework introduced in [7].

**Table 1:** Definitions of the elementary metrics used to formulate the evaluation metrics.

Metric	Definition	Description	Metric	Definition	Description
**TP**	P(Y=1,θ=1)	True Positive (correctly identified)	**FN**	P(Y=1,θ=0)	False Negative
**TN**	P(Y=0,θ=0)	True Negative (correctly rejected)	**FP**	P(Y=0,θ=1)	False Negative
**TPR**	TP/(TP + FN)	True Positive Rate	**TNR**	TN/(FP + TN)	True Negative Rate
**Precision**	TP/(TP + FP)	Positive Predictive Value	**Recall**	TP/(TP + FN)	True Positive Rate

**Table 2:** Definitions of the metrics used for classification evaluation. At the exception of Accuracy the other metrics are suited for imbalanced data.

Metric	Expression	Reference
**MCC**	$\frac{TP\times TN-FP\times FN}{\sqrt{\left(TP+FN)(TP+FP)(TN+FP)(TN+FN\right)}}$	[12]
**AUC**	Area under ROC Curve	[18]
**Accuracy**	(*TPR* + *TNR*)/2	[9]
***F*_1_**	$2/(\frac{1}{Recall}+\frac{1}{Precision})$	[9, 19, 20]

### 1.1 SVM for imbalanced learning

For a benchmark, we selected Support Vector Machine (SVM) for imbalanced data as a good method from the literature. SVM performs classification by finding the hyperplane (*wx* + *b*) that maximizes the margin between the two classes. However, there are situations where a nonlinear boundary can separate the groups more efficiently. SVM handles this by using a kernel function (nonlinear) to map the data into a high dimensional space. The performance of the SVM classifier mainly relies on the choice of kernel function and the tuning of various parameters in the kernel function The Gaussian radial basis function are among the popular kernels. For imbalanced data sets we typically use misclassification penalty per class. This is called class-weighted SVM, which minimizes the following program:

$$\begin{array}{c} \text{SVM-imba}\;\{\begin{array}{c} \min_{w,\xi \geq 0}\frac{{\left||w|\right|}^{2}}{2}+C^{+}\sum_{i:y_{i}=0}\xi_{i}+C^{-}\underset{i:y_{i}=1}{\sum \xi_{i}}\\ s.t\\ y_{i}\left(wx_{i}+b\right)\geq 1-\xi_{i}\;\forall i \end{array} \end{array}$$

where *ξ*_*i*_ is a positive slack variable such that if 0 < *ξ*_*i*_ < 1 then instance *i* is between margin and correct side of hyperplane and if *ξ*_*i*_ > 1 then instance *i* is misclassified. The parameters *C*^+^ and *C*^−^ are the slack penalties for positive and negative classes receptively.

**Fig 1:**
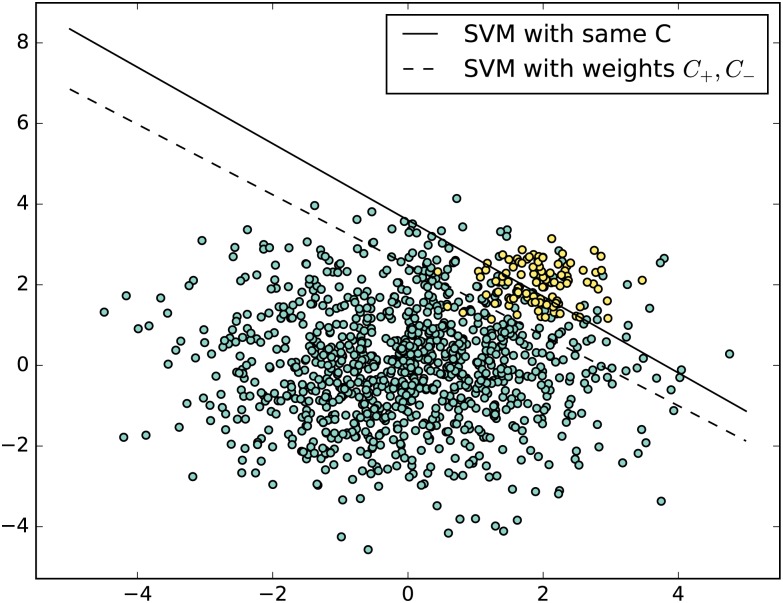
An illustration of the effect of introducing different weights in SVM to handle imbalance.

In this paper, we have used an imbalance SVM with the Gaussian kernel such that for two instances *x* and *x*′, we have *K*(*x*, *x*′) = *exp*(−*γ*||*x* − *x*′||^2^). The global model has three parameters *C*^+^, *C*^−^ and *γ*. Fig 1 gives an example of the effect of introducing two regularization weights on the classification results. The decision boundary is shifted towards the the majority class and hence the performance improved in this example.

We have conducted an experimental analysis to set the value of these parameters based on the training data. We used the rule of thumb suggested by Akbani et al. that the ratio $\frac{C^{+}}{C^{-}}$ is equal to the minority-to-majority class ratio [11].

The remainder of this paper is organized as follows. In Section 2, we describe a version of Support Vector Machines that handles imbalanced data. In Section 3, we propose an optimal classifier based on MCC metric. We show that it is consistent, i.e., it converges asymptotically to the theoretical optimal classifier. In the last section, we present and discuss the experimental results.

## 2 MCC metric for imbalanced data

### 2.1 MCC definition

The MCC metric has been first introduced by B.W. Matthews to assess the performance of protein secondary structure prediction [12]. Then, it becomes a widely used performance measure in biomedical research [13–17]. MCC and Area Under ROC Curve (AUC) have been chosen as the elective metric in the US FDA-led initiative MAQC-II that aims to reach a consensus on the best practices for development and validation of predictive models for personalized medicine [16].

Let X be the instance space, *X* a real valued random input vector, and *Y* ∈ {0, 1} a binary output variable, with joint distribution (X,Y)∼P. Let Θ be the space of classifiers Θ={θ:X↦[0,1]}. We define the quantities: π=P(Y=1), γ(θ)=P(θ=1) and TP(θ,P)=P(Y=1,θ=1). We define the conditional probability $\eta_{x}=P\left(Y=1|X=x\right)$.

The MCC can be seen as a discretization of the Pearson correlation for binary variables. In fact, given two *n*-vectors **x** = (*x*_1_, …, *x*_*n*_)^*t*^ and **y** = (*y*_1_, …, *y*_*n*_)^*t*^, recall that the sample linear correlation coefficient is given by

$$\begin{array}{c} r\left(x,y\right)=\frac{\sum_{i=1}^{n}\left(x_{i}-\bar{x}\right)\left(y_{i}-\bar{y}\right)}{\sqrt{\sum_{i=1}^{n}{\left(x_{i}-\bar{x}\right)}^{2}}\sqrt{\sum_{i=1}^{n}{\left(y_{i}-\bar{y}\right)}^{2}}} \end{array}$$

If **x** and **y** are binary, using some algebra, we have

$$\begin{array}{ccc} MCC(x,y)\equiv r(x,y) & = & \frac{n\times TP-(TP+FN)(TP+FP)}{\sqrt{\left(TP+FN)(TP+FP)(TN+FP)(TN+FN\right)}}\\ & = & \frac{TP\times TN-FP\times FN}{\sqrt{\left(TP+FN)(TP+FP)(TN+FP)(TN+FN\right)}} \end{array}$$

### 2.2 Suitability of MCC for imbalanced data

**Fig 2:**
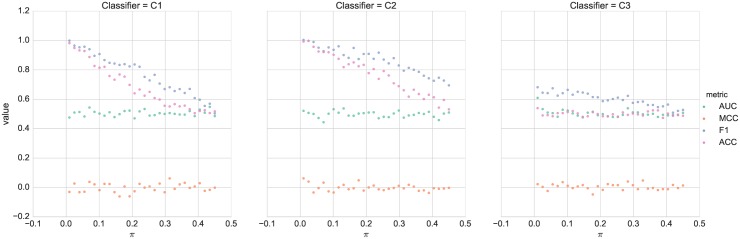
Performance comparison of the 3 classifiers described in Table 3.

In order to demonstrate the suitability of MCC for imbalanced data, we considered the following simulations: We generated 10000 random class labels {0, 1} such that the proportion of class 1 is equal to predefined value *π* < 0.5. We considered three basic classifiers: 1) *C*_1_: a classifier that generates stratified random prediction by respecting the training sets class distribution 2) *C*_2_: a classifier that always outputs 0, i.e., the class with the largest sample size, 3) *C*_3_: a classifier that generates random prediction uniformly. For this simulation, we compared the following metrics, MCC, AUC, Accuracy and *F*_1_ described in Table 2. We note that the three classifiers generate the labels without looking at the information carried by any feature vector. Therefore it is not expected that any of these classifiers should outperform the others. A good performance metric should indicate that these classifiers have comparable performance. Fig 2 summarizes the simulation results. The class proportion *π* is varied between 0 and 0.5. For each choice of *π*, 10000 labels and prediction values for the three classifiers are generated and the performances for the four metrics are computed. The accuracy and *F*_1_ metrics gave varying performances for classifiers *C*_1_ and *C*_2_ for the different choices of *π*. Thus these two metrics are sensitive to the data imbalance. The metric *F*_1_ also showed somewhat varying performance for classifier *C*_3_. On the other hand both metrics MCC and AUC have shown constant performance for the different classifiers. Therefore MCC and AUC are robust to data imbalance. The limitation of using AUC is that there is no explicit formula to compute AUC. On the other hand, MCC has a close form and it is very well suited to be used for building the optimal classifier for imbalanced data.

**Table 3:** Description of the three simple classifiers. They are used to evaluate the behavior of metrics in Table 2 for imbalanced data.

Classifiers	Description
*C*_1_	Generates random predictions by respecting the training set’s class distribution.
*C*_2_	Always predicts the most frequent label in the training set.
*C*_3_	Generates predictions uniformly at random.

### 2.3 Optimal consistent classifier for MCC metric

Matthews Correlation Coefficient (MCC) is defined in terms of True Positive (TP), True Negative (TN), False Positive (FP) and False Negative (FN). It can also be re-written in terms of TP, *γ* and *π* as follows:

$$\begin{array}{c} MCC\left(\theta \right)=\frac{TP\cdot TN-FP\cdot FN}{\sqrt{\left(TP+FP)(FP+FN)(TN+FP)(TN+FN\right)}}=\frac{TP-\gamma \pi }{\sqrt{\gamma (1-\gamma )\pi (1-\pi )}}. \end{array}$$

We recall that γ=P(θ=1) is and π=P(Y=1). If the small class is considered to have the label 1 than *π* corresponds to the minority class proportion. We quote here some of the remarks about the MCC metric as mentioned by Baldi et al. [21]:

- The MCC can be calculated using the confusion matrix.
- The calculation of the MCC metric uses the four quantities (TP, TN, FP and FN), which gives a better summary of the performance of classification algorithms.
- The MCC is not defined if any of the quantities *TP* + *FN*, *TP* + *FP*, *TN* + *FP*, or *TN* + *FN* is zero.
- MCC takes values in the interval [−1, 1], with 1 showing a complete agreement, −1 a complete disagreement, and 0 showing that the prediction was uncorrelated with the ground truth.

Theorems 1 and 2 provide the optimal form of the MCC classifier and its consistency, respectively. Since the optimal threshold δ* is dependent on TP* it cannot be directly used in Algorithm 1. Instead a grid search can be used for determining the optimal threshold.

We recall that the distribution P satisfies Assumption A (AA for short) if *P*(*η*_*x*_ ≺ *c*|*y* = 1) and *P*(*η*_*x*_ ≺ *c*|*y* = 0) are continuous for $c=\delta^{*}=\frac{TP+\gamma (\pi -2TP)}{2\gamma (1-\gamma )}$. We note that AA is verified in particular if the random variables (*η*_*x*_|*y* = 1) and (*η*_*x*_|*y* = 0) are continuous.

**Theorem 1.** ***(Optimal classifier for MCC metric)*** *Let* P *be a distribution on* X×[0,1] *that satisfies assumption A. The optimal binary classifier for the MCC metric is a thresholded classifier* θ*(*x*) = *sign*[(*TP* − *γπ*)(*η*_*x*_ − δ*)] *where the threshold* δ* *is defined by* $\delta^{*}=\frac{TP^{*}+\gamma \left(\pi -2\;TP^{*}\right)}{2\gamma (1-\gamma )}$.

The proof of the theorem involves the use of Frechet derivative that generalizes the notion of derivation to functions. It is therefore possible to obtain a close form of the optimal classifier. Theorem 1 ensures that the optimal classifier is either sign[(*η*_*x*_ − δ*)] or sign[−(*η*_*x*_ − δ*)] since the term (*TP* − *γπ*) is unknown before designing the classifier. The idea of the optimal classifier algorithm consists of finding the best classifiers among the set of classifiers sign[(*η*_*x*_ − *δ*)] and sign[−(*η*_*x*_ − *δ*)] for a certain constant *δ*. We note that both of these classifiers are among our space of classifiers Θ. Firstly, we divide the training set into two disjoint sets *S*_1_ and *S*_2_. Secondly, we estimate the conditional distribution *η*_*x*_ on *S*_1_ by using for example a regularized logistic regression. Thirdly, for each value of *δ*, we compute the *MCC* performance of the associated classifiers sign[(*η*_*x*_ − *δ*)] and sign[−(*η*_*x*_ − *δ*)] based on the set *S*_2_. Finally, we apply a grid search on *δ* to select the best classifier having the highest *MCC* performance.

The algorithm can be described as follows:

**Algorithm 1**: Algorithm for estimating the optimal MCC classifier.

1 Split the training set $S={\left\{\left(X_{i},Y_{i}\right)\right\}}_{i=1}^{n}$ into two sets *S*_1_ and *S*_2_

2 Estimate *η*_*x*_ using *S*_1_, define ${\hat{\theta_{1}}}_{\delta }=\text{sign}[({\hat{\eta }}_{x}-\delta )]$ and ${\hat{\theta_{2}}}_{\delta }=\text{sign}[-({\hat{\eta }}_{x}-\delta )]$

3 Compute $\hat{\delta }=\arg\max_{\delta \in (0,1)}\left\{MCC_{n}\left({\hat{\theta_{1}}}_{\delta }\right),MCC_{n}\left({\hat{\theta_{2}}}_{\delta }\right)\right\}$ on *S*_2_; where $MCC_{n}\left(\theta \right)=\frac{TP_{n}-\gamma_{n}\pi }{\sqrt{\gamma_{n}\left(1-\gamma_{n}\right)\pi \left(1-\pi \right)}}$ for classifier *θ*

4 If $MCC_{n}\left({\hat{\theta_{1}}}_{\hat{\delta }}\right)\geq MCC_{n}\left({\hat{\theta_{2}}}_{\hat{\delta }}\right)$ then return ${\hat{\theta_{1}}}_{\hat{\delta }}$, else return ${\hat{\theta_{2}}}_{\hat{\delta }}$

Another interesting property is to check the statistical consistency of the optimal MCC classifier. This property ensures that the estimated classifier converges in probability to the theoretical classifier. It gives asymptotic guarantees that the classifier gets closer to the theoretical best classifier as the size of training data increases.

**Theorem 2.** ***(Consistency of the optimal classifier)***. *The optimal classifier defined in Theorem 1 is consistent if the estimate* ${\hat{\eta }}_{x}$ *is obtained using a proper loss function* [22, 23].

The proofs of theorems 1 and 2 are provided in the supplemental material S1 File.

## 3 Results

### 3.1 Synthetic data

**Fig 3:**
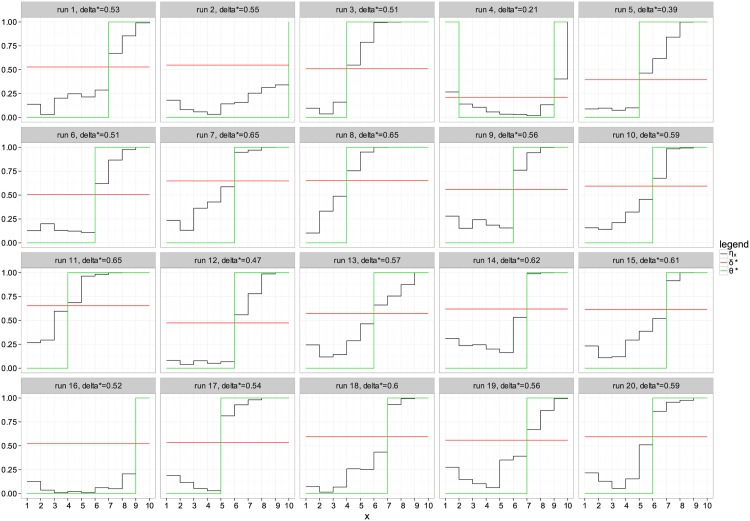
Optimal classifier for different simulations. The x-axis depcits the possible values in the feature space. The y-axis depicts probability values. δ*, shown in red, is the optimal derived threshold. The green curve depicts the optimal classifier obtained by exhaustive search maximizing MCC.

The optimality result given in Theorem 2 gives an interesting insight on designing classifier that could perform close to the optimal classifier. In order to validate the results, we considered a synthetic dataset similar to the one proposed in [6]. In this problem we know the space of all possible classifiers. Therefore we can exhaustively search for the optimal classifier and compare the obtained result with our finding in Theorem 2. We define a domain X={1,2,⋯,10}. The posterior distribution *μ*(*x*) is defined by generating a random series of value drawn from a uniform distribution on the interval [0, 1]. The conditional distribution is defined by $\eta_{x}=\frac{1}{1+exp(-wx)}$ where *w* is drawn from a standard Gaussian distribution N(0,1). The MCC classifier θ* is obtained using an exhaustive search over all 2^10^ possible classifiers. Fig 3 summarizes the simulation results for 20 random draw of the data. The black curves represent the distribution *η*_*x*_ for the different values in X. The green curves show that optimal MCC classifier obtained by a search across all possible classifiers. Each element in X can be either classified as negative (-1) or positive (1). Thus in total we have 2^10^ possible classifiers. the optimal classifier is obtained by searching the classifier that maximizes the performance measure MCC. The red curves denote the threshold obtained using the result in Theorem 1. The value of the threshold is shown in the header of each draw. We can clearly see that the derived threshold from Theorem 1 corresponds in all illustrated cases to the optimal classifier obtained by the exhaustive search. Therefore we can verify that the proposed optimal classifier is the same as obtained by an exhaustive search.

### 3.2 Real-world data

**Fig 4:**
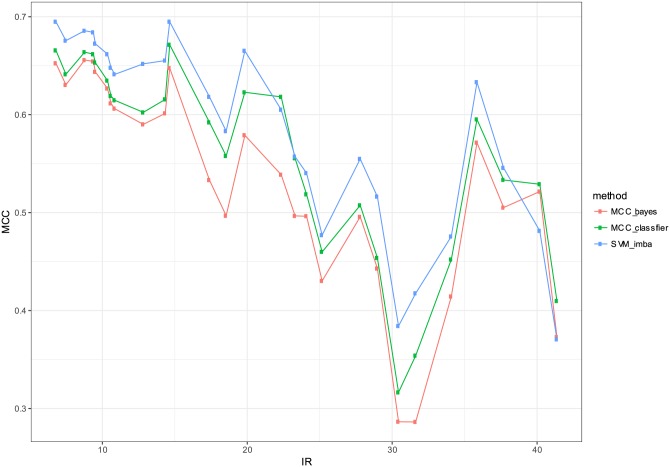
MCC performance comparison of the three classifiers (MCC-bayes, MCC-classifier, SVM-imba).

**Fig 5:**
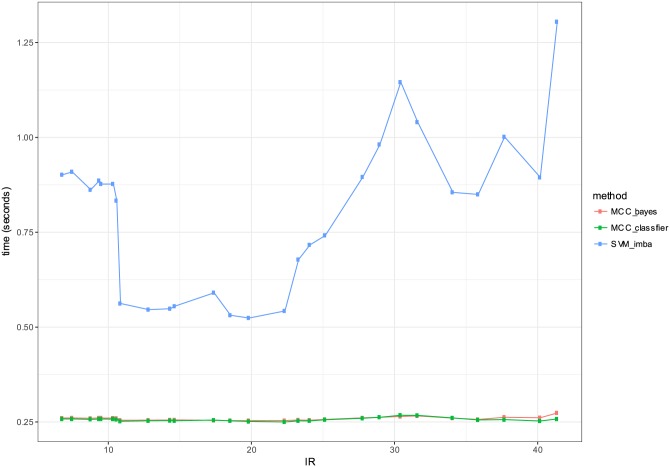
Comparison of the three evaluated classifiers in terms of computational efficiency (measured in seconds) of training phase.

In order to evaluate the performance of the proposed classifier, we considered real-world datasets that are publicly available. Table 4 summarizes the tested datasets. We collected 64 datasets that have been previously proposed for the evaluation of imbalanced data classification [24]. The summary table provides information on the number of examples, number of features, the imbalance ratio (IR) and the percentage of the minority class. The imbalance ratio (IR) is defined as the size ratio of the large class and the small class. In our comparison, we included three classifiers: 1) MCC-classifier: the proposed optimal classifier with the proposed threshold choice as in Algorithm 1, 2) MCC-bayes: the Bayes classifier with a default threshold choice *δ* = 0.5 [25]. 3) SVM-imba: imbalanced SVM classifier with Gaussian kernel that maximizes MCC metric. The data is split into training and testing sets. The training set is used for fitting and estimating hyper-parameters. The test set is only used for the evaluation of the classifiers. The previous procedure is repeated 10 times by randomly generating the train and test set in order to estimate the the means and standard deviations as depicted in the result tables. The estimate ${\hat{\eta }}_{x}$ is fitted based a regularized logistic regression. Tables 5 and 6 give the detailed performance results for each dataset respectively in terms of MCC and computation time. Table 7 presents the average performance of the three compared methods. SVM-imba outperforms our method by about 3% in terms of MCC. The proposed MCC-classifier was the best method for 24 datasets versus 31 datasets for SVM-imba, while SVM-bayes was the best classifier for 9 datasets. Similarly Table 8 presents the summary comparison of the training computation time for the three methods. MCC-classifier and MCC-bayes have a significant efficiency advantage compared with SVM-imba. Both methods are two to five folds faster than SVM-imba. This computational advantage is crucial when the size of training dataset becomes very large. Fig 4 gives a summary of the performance comparison. Although SVM-imba shows an overall better performance than MCC-classifier, this difference is not very large and the proposed method was able to be the winner in almost as many datasets as for SVM-imba. We think that the strength of SVM-imba is due to its ability to better handle strong non-linearity in the dataset using the high-dimensional mapping obtained by the Gaussian kernel. Moreover, SVM-imba had three tuning parameters namely the regularization parameters *C*^+^, *C*^−^ and the kernel parameter *σ*. These parameters are giving more capacity to SVM-imba compared with the proposed approach. In terms of computational efficiency, MCC-classifier is much faster to train than SVM-imba. Table of the algorithm assessed in terms of CPU during cross-validation of the training and testing are depicted in Fig 5. Our optimal MCC classifier is clearly more efficient than SVM and has a comparable efficiency to the plugin classifier. Therefore the introduced optimal classifier comes with some advantages: It gives a good trade-off between computational efficiency and performance. It provides also attractive theoretical properties such as consistency and optimality.

**Table 4:** Description of the 64 datasets used in the experimental section. Sample and feature sizes, imbalance ratio (IR) and small-class proportion *π*. The rows are sorted by imbalance ratio (IR).

Datasets	# samples	# features	IR	*π* in %
glass1	213	9	1.80	35.68
ecoli-0_vs_1	219	7	1.84	35.16
wisconsin	682	9	1.85	35.04
pima	767	8	1.87	34.81
iris0	149	4	2.04	32.89
glass0	213	9	2.09	32.39
yeast1	1483	8	2.46	28.93
haberman	305	3	2.77	26.56
vehicle2	845	18	2.88	25.80
vehicle1	845	18	2.89	25.68
vehicle3	845	18	2.99	25.09
glass-0-1-2-3_vs_4-5-6	213	9	3.18	23.94
vehicle0	845	18	3.27	23.43
ecoli1	335	7	3.35	22.99
new-thyroid1	214	5	5.11	16.36
new-thyroid2	214	5	5.11	16.36
ecoli2	335	7	5.44	15.52
segment0	2307	19	6.01	14.26
glass6	213	9	6.34	13.62
yeast3	1483	8	8.10	10.99
ecoli3	335	7	8.57	10.45
page-blocks0	5471	10	8.79	10.22
ecoli-0-3-4_vs_5	199	7	8.95	10.05
ecoli-0-6-7_vs_3-5	221	7	9.05	9.95
ecoli-0-2-3-4_vs_5	201	7	9.05	9.95
yeast-2_vs_4	513	8	9.06	9.94
glass-0-1-5_vs_2	171	9	9.06	9.94
ecoli-0-4-6_vs_5	202	6	9.10	9.90
yeast-0-3-5-9_vs_7-8	505	8	9.10	9.90
glass-0-4_vs_5	91	9	9.11	9.89
ecoli-0-1_vs_2-3-5	243	7	9.12	9.88
yeast-0-2-5-7-9_vs_3-6-8	1003	8	9.13	9.87
yeast-0-2-5-6_vs_3-7-8-9	1003	8	9.13	9.87
ecoli-0-2-6-7_vs_3-5	223	7	9.14	9.87
ecoli-0-3-4-6_vs_5	204	7	9.20	9.80
ecoli-0-3-4-7_vs_5-6	256	7	9.24	9.77
yeast-0-5-6-7-9_vs_4	527	8	9.33	9.68
ecoli-0-6-7_vs_5	219	6	9.95	9.13
vowel0	987	13	10.09	9.02
glass-0-1-6_vs_2	191	9	10.24	8.90
ecoli-0-1-4-7_vs_2-3-5-6	335	7	10.55	8.66
glass-0-6_vs_5	107	9	10.89	8.41
led7digit-0-2-4-5-6-7-8-9_vs_1	442	7	10.95	8.37
ecoli-0-1_vs_5	239	6	10.95	8.37
glass-0-1-4-6_vs_2	204	9	11.00	8.33
glass2	213	9	11.53	7.98
cleveland-0_vs_4	172	13	12.23	7.56
ecoli-0-1-4-7_vs_5-6	331	6	12.24	7.55
ecoli-0-1-4-6_vs_5	279	6	12.95	7.17
shuttle-c0-vs-c4	1828	9	13.86	6.73
yeast-1_vs_7	458	7	14.27	6.55
glass4	213	9	15.38	6.10
ecoli4	335	7	15.75	5.97
page-blocks-1-3_vs_4	471	10	15.82	5.94
glass-0-1-6_vs_5	183	9	19.33	4.92
yeast-1-4-5-8_vs_7	692	8	22.07	4.34
glass5	213	9	22.67	4.23
yeast-2_vs_8	481	8	23.05	4.16
shuttle-c2-vs-c4	128	9	24.60	3.91
yeast4	1483	8	28.08	3.44
yeast-1-2-8-9_vs_7	946	8	30.53	3.17
yeast5	1483	8	32.70	2.97
ecoli-0-1-3-7_vs_2-6	280	7	39.00	2.50
yeast6	1483	8	41.37	2.36

**Table 5:** Performance comparison in terms of MCC metric. The three compared classifiers are MCC-bayes, MCC-classifier and SVM-imba. The rows are sorted by imbalance ratio IR. ± depicts one SD.

Datasets	MCC-bayes	MCC-classifier	SVM-imba	IR
glass1	0.16 ± 0.1	0.15 ± 0.11	0.45 ± 0.09	1.80
ecoli-0_vs_1	0.96 ± 0.1	0.97 ± 0.22	0.97 ± 0.18	1.84
wisconsin	0.92 ± 0.16	0.93 ± 0.13	0.92 ± 0.09	1.85
pima	0.46 ± 0.07	0.48 ± 0.1	0.45 ± 0.13	1.87
iris0	1 ± 0.08	0.99 ± 0.09	1 ± 0.12	2.04
glass0	0.4 ± 0.08	0.43 ± 0.09	0.59 ± 0.09	2.09
yeast1	0.38 ± 0.06	0.35 ± 0.09	0.4 ± 0.08	2.46
haberman	0.19 ± 0.12	0.2 ± 0.07	0.25 ± 0.09	2.77
vehicle2	0.89 ± 0.12	0.92 ± 0.12	0.95 ± 0.09	2.88
vehicle1	0.43 ± 0.11	0.46 ± 0.06	0.62 ± 0.09	2.89
vehicle3	0.41 ± 0.16	0.42 ± 0.11	0.58 ± 0.14	2.99
glass-0-1-2-3_vs_4-5-6	0.78 ± 0.11	0.81 ± 0.09	0.8 ± 0.08	3.18
vehicle0	0.9 ± 0.12	0.92 ± 0.06	0.93 ± 0.07	3.27
ecoli1	0.68 ± 0.23	0.67 ± 0.11	0.69 ± 0.08	3.35
new-thyroid1	0.94 ± 0.1	0.94 ± 0.12	0.94 ± 0.08	5.11
new-thyroid2	0.94 ± 0.03	0.96 ± 0.04	0.93 ± 0.03	5.11
ecoli2	0.68 ± 0.05	0.68 ± 0.06	0.82 ± 0.03	5.44
segment0	0.99 ± 0.08	0.99 ± 0.09	0.99 ± 0.1	6.01
glass6	0.8 ± 0.1	0.83 ± 0.09	0.82 ± 0.1	6.34
yeast3	0.72 ± 0.09	0.69 ± 0.15	0.72 ± 0.06	8.10
ecoli3	0.49 ± 0.11	0.47 ± 0.07	0.53 ± 0.07	8.57
page-blocks0	0.68 ± 0.07	0.69 ± 0.05	0.8 ± 0.05	8.79
ecoli-0-3-4_vs_5	0.73 ± 0.11	0.75 ± 0.11	0.75 ± 0.15	8.95
ecoli-0-6-7_vs_3-5	0.62 ± 0.18	0.67 ± 0.16	0.71 ± 0.09	9.05
ecoli-0-2-3-4_vs_5	0.78 ± 0.18	0.79 ± 0.16	0.77 ± 0.24	9.05
glass-0-1-5_vs_2	0.09 ± 0.39	0.03 ± 0.16	0.28 ± 0.17	9.06
yeast-2_vs_4	0.72 ± 0.32	0.74 ± 0.3	0.66 ± 0.2	9.06
ecoli-0-4-6_vs_5	0.75 ± 0.45	0.78 ± 0.22	0.82 ± 0.11	9.10
yeast-0-3-5-9_vs_7-8	0.32 ± 0.15	0.37 ± 0.12	0.28 ± 0.1	9.10
glass-0-4_vs_5	0.64 ± 0.16	0.7 ± 0.14	0.76 ± 0.18	9.11
ecoli-0-1_vs_2-3-5	0.77 ± 0.28	0.78 ± 0.2	0.68 ± 0.13	9.12
yeast-0-2-5-6_vs_3-7-8-9	0.52 ± 0.34	0.5 ± 0.18	0.54 ± 0.22	9.13
yeast-0-2-5-7-9_vs_3-6-8	0.76 ± 0.12	0.8 ± 0.08	0.76 ± 0.09	9.13
ecoli-0-2-6-7_vs_3-5	0.68 ± 0.07	0.69 ± 0.09	0.63 ± 0.05	9.14
ecoli-0-3-4-6_vs_5	0.7 ± 0.01	0.77 ± 0.03	0.78 ± 0	9.20
ecoli-0-3-4-7_vs_5-6	0.69 ± 0.07	0.66 ± 0.07	0.67 ± 0.08	9.24
yeast-0-5-6-7-9_vs_4	0.46 ± 0.03	0.49 ± 0.04	0.39 ± 0.04	9.33
ecoli-0-6-7_vs_5	0.77 ± 0.05	0.79 ± 0.05	0.72 ± 0.07	9.95
vowel0	0.8 ± 0.03	0.84 ± 0.03	0.99 ± 0.01	10.09
glass-0-1-6_vs_2	0.27 ± 0.1	0.03 ± 0.11	0.37 ± 0.1	10.24
ecoli-0-1-4-7_vs_2-3-5-6	0.71 ± 0.07	0.71 ± 0.05	0.64 ± 0.05	10.55
glass-0-6_vs_5	0.55 ± 0.01	0.87 ± 0.01	0.78 ± 0.01	10.89
led7digit-0-2-4-5-6-7-8-9_vs_1	0.78 ± 0.01	0.78 ± 0.01	0.67 ± 0.01	10.95
ecoli-0-1_vs_5	0.83 ± 0.12	0.79 ± 0.09	0.78 ± 0.12	10.95
glass-0-1-4-6_vs_2	0.09 ± 0.03	0.05 ± 0.02	0.29 ± 0.02	11.00
glass2	0.18 ± 0.05	0.11 ± 0.04	0.3 ± 0.04	11.53
cleveland-0_vs_4	0.68 ± 0.03	0.59 ± 0.03	0.58 ± 0.02	12.23
ecoli-0-1-4-7_vs_5-6	0.8 ± 0.03	0.77 ± 0.04	0.72 ± 0.03	12.24
ecoli-0-1-4-6_vs_5	0.66 ± 0.05	0.7 ± 0.05	0.76 ± 0.01	12.95
shuttle-c0-vs-c4	1 ± 0.02	1 ± 0.01	0.99 ± 0.01	13.86
yeast-1_vs_7	0.26 ± 0.06	0.35 ± 0.07	0.26 ± 0.05	14.27
glass4	0.45 ± 0.05	0.42 ± 0.03	0.78 ± 0.03	15.38
ecoli4	0.83 ± 0.13	0.81 ± 0.06	0.73 ± 0.12	15.75
page-blocks-1-3_vs_4	0.66 ± 0.09	0.66 ± 0.11	0.79 ± 0.07	15.82
glass-0-1-6_vs_5	0.5 ± 0.04	0.74 ± 0.03	0.64 ± 0.02	19.33
yeast-1-4-5-8_vs_7	0 ± 0.15	0 ± 0.18	0.09 ± 0.06	22.07
glass5	0.58 ± 0.01	0.77 ± 0.01	0.67 ± 0.08	22.67
yeast-2_vs_8	0.71 ± 0.2	0.72 ± 0.13	0.71 ± 0.07	23.05
shuttle-c2-vs-c4	0.91 ± 0.07	0.87 ± 0.06	0.91 ± 0.06	24.60
yeast4	0.29 ± 0.05	0.24 ± 0.08	0.32 ± 0.1	28.08
yeast-1-2-8-9_vs_7	0.1 ± 0.04	0.17 ± 0.04	0.16 ± 0.03	30.53
yeast5	0.47 ± 0.05	0.54 ± 0.08	0.67 ± 0.06	32.70
ecoli-0-1-3-7_vs_2-6	0.67 ± 0.08	0.65 ± 0.1	0.59 ± 0.06	39.00
yeast6	0.37 ± 0.16	0.41 ± 0.1	0.37 ± 0.09	41.37

**Table 6:** Comparison of training time (in seconds). The three classifiers are MCC-bayes, MCC-classifier and SVM-imba. The rows are sorted by imbalance ratio IR. ± depicts one SD.

Datasets	MCC-bayes	MCC-classifier	SVM-imba	IR
glass1	0.14 ± 0.15	0.16 ± 0.09	0.43 ± 0.11	1.80
ecoli-0_vs_1	0.91 ± 0.09	0.93 ± 0.09	0.96 ± 0.15	1.84
wisconsin	0.93 ± 0.14	0.92 ± 0.09	0.93 ± 0.09	1.85
pima	0.46 ± 0.07	0.45 ± 0.1	0.45 ± 0.09	1.87
iris0	0.97 ± 0.09	0.97 ± 0.11	1 ± 0.16	2.04
glass0	0.39 ± 0.14	0.45 ± 0.09	0.58 ± 0.1	2.09
yeast1	0.37 ± 0.12	0.38 ± 0.07	0.4 ± 0.06	2.46
haberman	0.17 ± 0.13	0.16 ± 0.11	0.23 ± 0.11	2.77
vehicle2	0.91 ± 0.09	0.9 ± 0.11	0.94 ± 0.16	2.88
vehicle1	0.45 ± 0.14	0.44 ± 0.12	0.65 ± 0.11	2.89
vehicle3	0.38 ± 0.11	0.4 ± 0.12	0.57 ± 0.07	2.99
glass-0-1-2-3_vs_4-5-6	0.8 ± 0.13	0.76 ± 0.17	0.83 ± 0.13	3.18
vehicle0	0.92 ± 0.12	0.91 ± 0.1	0.93 ± 0.07	3.27
ecoli1	0.69 ± 0.05	0.67 ± 0.15	0.69 ± 0.09	3.35
new-thyroid1	0.95 ± 0.13	0.92 ± 0.1	0.94 ± 0.09	5.11
new-thyroid2	0.94 ± 0.07	0.94 ± 0.05	0.96 ± 0.02	5.11
ecoli2	0.64 ± 0.08	0.65 ± 0.07	0.82 ± 0.05	5.44
segment0	0.99 ± 0.07	0.99 ± 0.07	0.99 ± 0.06	6.01
glass6	0.84 ± 0.12	0.83 ± 0.13	0.83 ± 0.08	6.34
yeast3	0.71 ± 0.11	0.72 ± 0.18	0.73 ± 0.08	8.10
ecoli3	0.49 ± 0.07	0.49 ± 0.08	0.53 ± 0.06	8.57
page-blocks0	0.7 ± 0.08	0.7 ± 0.07	0.81 ± 0.06	8.79
ecoli-0-3-4_vs_5	0.74 ± 0.21	0.71 ± 0.13	0.8 ± 0.19	8.95
ecoli-0-6-7_vs_3-5	0.65 ± 0.04	0.58 ± 0.06	0.63 ± 0.1	9.05
ecoli-0-2-3-4_vs_5	0.75 ± 0.18	0.77 ± 0.16	0.8 ± 0.19	9.05
glass-0-1-5_vs_2	-0.04 ± 0.25	-0.02 ± 0.21	0.41 ± 0.17	9.06
yeast-2_vs_4	0.7 ± 0.3	0.72 ± 0.23	0.64 ± 0.18	9.06
ecoli-0-4-6_vs_5	0.71 ± 0.4	0.66 ± 0.35	0.78 ± 0.27	9.10
yeast-0-3-5-9_vs_7-8	0.41 ± 0.11	0.41 ± 0.11	0.33 ± 0.09	9.10
glass-0-4_vs_5	0.69 ± 0.16	0.74 ± 0.19	0.84 ± 0.22	9.11
ecoli-0-1_vs_2-3-5	0.65 ± 0.17	0.66 ± 0.22	0.63 ± 0.14	9.12
yeast-0-2-5-6_vs_3-7-8-9	0.54 ± 0.46	0.52 ± 0.34	0.52 ± 0.21	9.13
yeast-0-2-5-7-9_vs_3-6-8	0.81 ± 0.04	0.81 ± 0.06	0.77 ± 0.06	9.13
ecoli-0-2-6-7_vs_3-5	0.64 ± 0.09	0.66 ± 0.06	0.6 ± 0.08	9.14
ecoli-0-3-4-6_vs_5	0.7 ± 0.04	0.68 ± 0.04	0.79 ± 0	9.20
ecoli-0-3-4-7_vs_5-6	0.68 ± 0.07	0.71 ± 0.06	0.69 ± 0.09	9.24
yeast-0-5-6-7-9_vs_4	0.45 ± 0.02	0.45 ± 0.04	0.4 ± 0.05	9.33
ecoli-0-6-7_vs_5	0.72 ± 0.03	0.69 ± 0.06	0.69 ± 0.03	9.95
vowel0	0.84 ± 0.03	0.82 ± 0.03	0.99 ± 0.01	10.09
glass-0-1-6_vs_2	0.13 ± 0.09	0.06 ± 0.11	0.3 ± 0.09	10.24
ecoli-0-1-4-7_vs_2-3-5-6	0.72 ± 0.04	0.66 ± 0.04	0.7 ± 0.02	10.55
glass-0-6_vs_5	0.51 ± 0.01	0.59 ± 0	0.72 ± 0	10.89
led7digit-0-2-4-5-6-7-8-9_vs_1	0.72 ± 0.01	0.74 ± 0.02	0.58 ± 0.01	10.95
ecoli-0-1_vs_5	0.76 ± 0.12	0.8 ± 0.33	0.74 ± 0.18	10.95
glass-0-1-4-6_vs_2	0.17 ± 0.02	0.05 ± 0.02	0.38 ± 0.02	11.00
glass2	0.1 ± 0.06	0.19 ± 0.04	0.27 ± 0.04	11.53
cleveland-0_vs_4	0.69 ± 0.03	0.66 ± 0.03	0.64 ± 0.02	12.23
ecoli-0-1-4-7_vs_5-6	0.7 ± 0.04	0.72 ± 0.06	0.64 ± 0.03	12.24
ecoli-0-1-4-6_vs_5	0.73 ± 0.04	0.75 ± 0.03	0.78 ± 0.02	12.95
shuttle-c0-vs-c4	0.99 ± 0.02	0.98 ± 0.02	0.99 ± 0.01	13.86
yeast-1_vs_7	0.22 ± 0.06	0.25 ± 0.07	0.29 ± 0.05	14.27
glass4	0.32 ± 0.05	0.35 ± 0.04	0.63 ± 0.05	15.38
ecoli4	0.83 ± 0.06	0.76 ± 0.08	0.76 ± 0.11	15.75
page-blocks-1-3_vs_4	0.58 ± 0.1	0.54 ± 0.11	0.78 ± 0.06	15.82
glass-0-1-6_vs_5	0.52 ± 0.04	0.67 ± 0.03	0.65 ± 0.02	19.33
yeast-1-4-5-8_vs_7	0 ± 0.15	-0.01 ± 0.16	0.09 ± 0.06	22.07
glass5	0.47 ± 0.01	0.61 ± 0.01	0.65 ± 0.08	22.67
yeast-2_vs_8	0.62 ± 0.15	0.61 ± 0.15	0.62 ± 0.09	23.05
shuttle-c2-vs-c4	0.84 ± 0.09	0.72 ± 0.07	0.86 ± 0.07	24.60
yeast4	0.25 ± 0.11	0.27 ± 0.13	0.31 ± 0.12	28.08
yeast-1-2-8-9_vs_7	0.15 ± 0.03	0.11 ± 0.02	0.17 ± 0.04	30.53
yeast5	0.46 ± 0.11	0.47 ± 0.1	0.67 ± 0.05	32.70
ecoli-0-1-3-7_vs_2-6	0.69 ± 0.08	0.69 ± 0.08	0.65 ± 0.07	39.00
yeast6	0.28 ± 0.19	0.45 ± 0.12	0.35 ± 0.09	41.37

**Table 7:** MCC average performance over the 64 datasets for the three compared classifiers.

MCC-bayes	MCC-classifier	SVM-imba
0.60 ± 0.29	0.62 ± 0.29	0.65 ± 0.24

**Table 8:** Computation time average (in seconds) over the 64 datasets for the three compared classifiers.

MCC-bayes	MCC-classifier	SVM-imba
0.22 ±0.022	0.22 ± 0.027	0.83 ± 1.16

In order to have a closer look at the performance of the proposed classifier as a function of the imbalance ratio we grouped the datasets that have IRs within intervals. The IR ranges are defined as follows: Datasets with IRs in the interval [*ir*, *ir* + 10], *ir* = 1, …, 40 are grouped and their MCC average performance is computed. Fig 4 presents the average results as a function of IR. SVM-imba has a slightly better performance than MCC-classifier for low IR. However it can be seen from the figure that high IR MCC-classifier has better performance. A similar analysis is performed for the training computational time. Fig 5 shows the average training time as a function of IR. It shows clearly that MCC-classifier and MCC-bayes outperforms SVM-imba by one to five folds in terms of computational time.

## 4 Conclusion

In this paper we proposed a new method for the classification of imbalanced data based on MCC metric. We showed the suitability of MCC for imbalanced data. We used Frechet derivative approach for devising the optimal form of the classifier. We showed that the obtained classifier has a sign form. As the threshold is dependent on the *TP* we derived an algorithm for estimating the classifier from training data. In the experimental analysis we showed, using simulated data, that the proposed classifier is indeed optimal in the space of all possible classifiers. We benchmarked our algorithm, MCC-classifier, on a large number of publicly available datasets, with respect to imbalanced SVM and MCC-bayes. We showed that our algorithm provides a good trade-off between performance in terms of MCC and computational efficiency in terms of training time. As future work, we would like to further explore the space of metrics suited for imbalanced data that can lead to constant threshold. Thus it will be possible to devise more efficient algorithms for imbalanced data.

## Supporting information

S1 File S1_File.pdf. This file contains the proofs of theorem 1 and theorem 2.
(PDF)
