# Supplementary material for: Optimal classifier for imbalanced data using Matthews Correlation Coefficient metric
Source: PLoS One. 2017 Jun 2;12(6):e0177678. doi: 10.1371/journal.pone.0177678 (PMC5456046; doi:10.1371/journal.pone.0177678)
Supplement: S1 File — This file contains the proofs of theorem 1 and theorem 2. (PDF) [file pone.0177678.s001.pdf]

Supplementary

1 General Optimality Results for Classifiers

We introduce here some general results that can be used for many categories of classifier.

Let X be a metric space with a measure $\mu(x)$, $F = \{f : .X \to R\}$ the set of real function or real classifier on X, let $G : F \to R$ be a functional on F and let $S \subset F$ be a set.

Consider the following optimization problem

$$P : \quad G^* = \max_{f \in \mathcal{F}} \mathcal{G}(f) \quad s.t. \qquad f \in S$$

A relaxation of P has the following form

$$P_R : \quad G_R^* = \max_{f \in \mathcal{F}} \mathcal{G}_{\mathcal{R}}(f) \quad s.t. \qquad f \in S_R$$

where $G_R : F \to R$ be a functional on F with $G_R \geq G$ on S and $S \subset S_R \subset F$.

Lemma 1. *(Relaxation lemma*

i) $G_R^* \geq G^*$.

ii) If P_R is infeasible, then so is P.

iii) If the problem P_R has an optimal solution $f_R^* \in S$ for which $G_R(f_R^*) = G(f_R^*)$, then (f_R^*) is an optimal solution to P as well.

Proof. i) By definition of the maximum, $G_R(f_R^*) \geqslant G_R(f) \quad \forall \quad f \in S_R$. In particular $G_R(f_R^*) \geqslant G_R(f) \quad \forall \quad f \in S$ and more particularly $G_R(f_R^*) \geqslant G(f) \quad \forall \quad f \in S$. So $G_R(f_R^*) \geqslant G(f^*) \quad \forall \quad f \in S$ and $G_R^* \geq G^*$.

ii) if S_R is empty then S is also empty

iii) Note that $G(f_R^*) = G_R(f_R^*) \geq G_R(f) \geq G(f) \forall \quad f \in S$. □

Lemma 2. *(Optimality conditions)*

Assume S is a convex set and G is Freshet derivative, i.e differentiable.

i) If $f*$ is a local minimum of P then $< \nabla \mathcal{G}(f^*), f^* - f > \quad \geq 0 \qquad \forall f \in S$

ii) if G is concave then condition i) is necessary and sufficient, and $f*$ is a global maximum

Proof. i) Since G is differentiable and S is convex, condition i) is exactly the first order optimality condition.

ii) condition i) is the necessary and sufficient conditions optimality condition for a convex problem.i.e the objective function is concave for a maximization problem and the admissible set is convex. □

We denote by $\Theta = \{f : \mathcal{X} \to [0, 1]\}$, i.e. the set of all classifiers. We consider the optimization problem

$$PL : \quad f^* = arg \max_{f \in \mathcal{F}} \mathcal{G}(f) \quad s.t. \qquad f \in \Theta$$

Lemma 3. *(Optimal classifier condition)*
The optimal solution of PL verifies

$$\int\limits_{x\in\mathcal{X}} [\nabla\mathcal{G}(f^*)]_x\,(f^*(x)\mu(x)dx \geq \int\limits_{x\in\mathcal{X}} [\nabla\mathcal{G}(f^*)]_x\,(f(x)\mu(x)dx \qquad \forall f \in \Theta$$

Proof. It is evident that Θ is convex. By applying lemma (Optimality conditions), the optimal solution of PL verifies

$$< \nabla\mathcal{G}(f^*), f^* - f > \quad \geq 0 \qquad \forall f \in \Theta$$

□

Thus

$$\int\limits_{x\in\mathcal{X}} [\nabla\mathcal{G}(f^*)]_x\,(f^*(x)dx \geq \int\limits_{x\in\mathcal{X}} [\nabla\mathcal{G}(f^*)]_x\,(f(x)dx \qquad \forall f \in \Theta$$

Theorem 4. *(Optimal classifier solution)*
The optimal solution f^* of PL verifies:

$$\textit{sign}\left([\nabla\mathcal{G}(f^*)]_x\right) = f(x*),\ \textit{almost everywhere}$$

where

$$\text{sign}(t) = \begin{cases} 1 & t \geq 0 \\ 0 & t < 0 \end{cases}$$

Proof. We will prove by contradiction. Suppose that

$$\text{sign}\left([\nabla\mathcal{G}(f^*)]_x\right) \neq f*(x),$$

So there exists $S_+, S_- \subset X$ two sets in X such that:
a)

$$\left([\nabla\mathcal{G}(f^*)]_x\right) > 0, \text{sign}\left([\nabla\mathcal{G}(f^*)]_x\right) = 1 \text{ and } f^*(x) < 1, \forall x \in S_+$$

b)

$$\left([\nabla\mathcal{G}(f^*)]_x\right) < 0, \text{sign}\left([\nabla\mathcal{G}(f^*)]_x\right) = 0 \text{ and } f^*(x) > 0, \forall x \in S_-$$

Lemma (Optimality conditions) can be applied for f=sign$\left([\nabla\mathcal{G}(f^*)]_x\right)$ because sign$\left([\nabla\mathcal{G}(f^*)]_x\right) \in \Theta$
Thus

$$\int\limits_{x\in\mathcal{X}} [\nabla\mathcal{G}(f^*)]_x\,(f^*(x)\mu(x)dx \geq \int\limits_{x\in\mathcal{X}} [\nabla\mathcal{G}(f^*)]_x\,\text{sign}\left([\nabla\mathcal{G}(f^*)]_x\right)\mu(x)dx$$

So

$$\int_{x\in\mathcal{X}} [\nabla\mathcal{G}(f^*)]_x \left(f^*(x) - sign\left([\nabla\mathcal{G}(f^*)]_x\right) \mu(x)dx \geq 0\right.$$

We will split the above integral into three parts as follows:

$$\int_{x\in\mathcal{X}\setminus S_+\sqcup S_-} [\nabla\mathcal{G}(f^*)]_x \left((f^*(x) - sign\left([\nabla\mathcal{G}(f^*)]_x\right)\right)\mu(x)dx$$
$$+ \int_{x\in S_+} [\nabla\mathcal{G}(f^*)]_x \left((f^*(x) - \text{sign}\left([\nabla\mathcal{G}(f^*)]_x\right)\right) \mu(x)dx$$
$$+ \int_{x\in S_-} [\nabla\mathcal{G}(f^*)]_x \left((f^*(x) - \text{sign}\left([\nabla\mathcal{G}(f^*)]_x\right)\right) \mu(x)dx \geq 0$$

-The first integral is null because for every $x \in \mathcal{X}\setminus S_+ \sqcup S_-$, sign $\left([\nabla\mathcal{G}(f^*)]_x\right) = f(x*)$,

-each element of the second integral is negative because $[\nabla\mathcal{G}(f^*)] > 0, f^*(x) < sign\left([\nabla\mathcal{G}(f^*)]_x\right))$. So the value of this integral is not greater than 0

-each element of the second integral is negative because $[\nabla\mathcal{G}(f^*)] < 0, f^*(x) > sign\left([\nabla\mathcal{G}(f^*)]_x\right))$. So the value of this integral is also not greater than 0.

Thus the sum of the two last integrals is positive however the value of each one is negative. Hence the value of each one is null.

Concerning the second integral, each term is negative and the value is null. So necessary the set S_+ is a zero measure set, i.e. $\mu(S_+) = 0$.

Similarly, we prove that the set S_- is a zero measure set, ie $\mu(S_-) = 0$. Since the measure $\mu(x)$ is additive and S_+ and S_- are disjoint, we have $\mu(S_+ \sqcup S_-) = 0$.Finally we conclude that

$$\text{sign}\left([\nabla\mathcal{G}(f^*)]_x\right) = f(x*), \text{ almost everywhere}$$

□

We denote by $\Theta_{01} = \{f : X \to \{0, 1\}$, i.e. the set of all binary classifiers. We consider the optimization problem

$$P01 : \quad f^* = arg \max_{f\in\mathcal{F}} \mathcal{G}(f) \quad s.t. \qquad f \in \Theta_{01}$$

Lemma 5. *(Optimal binary classifier solution)*

The optimal solution $f*$ of $P01$ verifies:

$$\text{sign}\left([\nabla\mathcal{G}(f^*)]_x\right) = f(x^*) \text{ almost everywhere}$$

Proof. Problem PL is a relaxation of $P01$ because the feasible set of PL extends that of $P01$. By lemma (relaxation) and since the optimal solution of PL is binary, we conclude that the optimal solution of PL is also an optimal solution of $P01$. So $arg \max_{f\in\mathcal{F}} \mathcal{G}(f) \quad s.t. \qquad f \in \Theta_{01} = \text{sign}\left([\nabla\mathcal{G}(f^*)]_x\right)$ □

2 MCC Metric

(from wikipedia) The Matthews correlation coefficient is used in machine learning as a measure of the quality of binary (two-class) classifications, introduced by biochemist

Brian W. Matthews in 1975.[1] It takes into account true and false positives and negatives and is generally regarded as a balanced measure which can be used even if the classes are of very different sizes.

$$MCC = \frac{TP \cdot TN - FP \cdot FN}{\sqrt{(TP+FP)(FP+FN)(TN+FP)(TN+FN)}}$$

Using the chosen notation the MCC metric can be simplified as follow

$$\mathcal{L}(f) = MCC = \frac{TP - \gamma\pi}{\sqrt{\gamma(1-\gamma)\pi(1-\pi)}}$$

2.1 Optimal MCC classifier

In order to define the optimal classifier, without loss of generality we look for the Frechet derivative of $\mathcal{L}(f) = MCC^2$

$$[\nabla \mathcal{L}(f)]_x = \frac{2(TP-\gamma\pi)\mu(x)}{\pi(1-\pi)\gamma(1-\gamma)} \left[\eta_x - \frac{TP+\gamma(\pi-2TP)}{2\gamma(1-\gamma)}\right]$$

i) if $TP > \gamma\pi$ then the optimal classifier takes the form $\theta^*(x) = \text{sign}(\eta_x - \delta^*)$
ii) if $TP < \gamma\pi$ then the optimal classifier takes the form $\theta^*(x) = \text{sign}(\delta^* - \eta_x)$

$$where \qquad \delta^* = \frac{TP + \gamma(\pi - 2TP)}{2\gamma(1-\gamma)}$$

Proof. Both results are derived from lemma 5 (Optimal binary classifier solution) . □

2.2 Consistency for the MCC metric

We will write the MCC metric as a function of (TPR, TNR, π) . We note that $TP = \pi TPR$ and $\gamma = \pi TPR + (1-\pi)(1-TNR)$

So $TP - \gamma\pi = \pi TPR - \gamma\pi = \pi(TPR - \gamma)$
$= \pi(1-\pi)(TPR + TNR - 1)$
Thus

$$MCC = \frac{\sqrt{\pi(1-\pi)}(TPR + TNR - 1)}{\sqrt{(\pi TPR + (1-\pi)(1-TNR))}\sqrt{(\pi(1-TPR) + (1-\pi)TNR}}$$

So $MCC = \psi(TPR, TPN, \pi)$ where

$$\psi(u, v, p) = (u + v - 1)\sqrt{\frac{p(1-p)}{[pu + (1-p)(1-v)][p(1-u) + (1-p)v]}}$$

We note that $\psi(u, v, p)$ is continuous in each argument.

1. Suppose Assumption A (AA for short) is valid for the data, ie $P(\eta_x \prec c|y=1)$ and $P(\eta_x \prec c|y=0)$ are continuous for $c = \delta^* = \frac{TP+\gamma(\pi-2TP)}{2\gamma(1-\gamma)}$. We note that AA is verified if the random variables $(\eta_x|y=1)$ and $(\eta_x|y=0)$ are continuous.

2. According to the work of Narasimhan et al. [1], under Assumption A, algorithm 1 is consistent since the optimal classifier is threshold and the function $\psi(u, v, p)$ is continuous

References

[1] Narasimhan H, Vaish R, Agarwal S. On the Statistical Consistency of Plug-in Classifiers for Non-decomposable Performance Measures. In: Ghahramani Z, Welling M, Cortes C, Lawrence ND, Weinberger KQ, editors. Advances in Neural Information Processing Systems 27. Curran Associates, Inc.; 2014. p. 1493–1501. Available from: http://papers.nips.cc/paper/5504-on-the-statistical-consistency-of-plug-in-classifiers-for-non-decomposa[illegible] pdf. 2
